# Supplementary material for: Shared Treg dysregulation underlies psoriasis vulgaris and atopic dermatitis through the PHF19-PRC2 epigenetic axis
Source: Front Immunol. 2026 Aug 20;17:1932788. doi: 10.3389/fimmu.2026.1932788 (PMC13538097; doi:10.3389/fimmu.2026.1932788)
Supplement: Supplementary file 3 [file DataSheet3.zip › Additional files/Additional file 8/13.DPYSL2.leaveoneout.pdf]

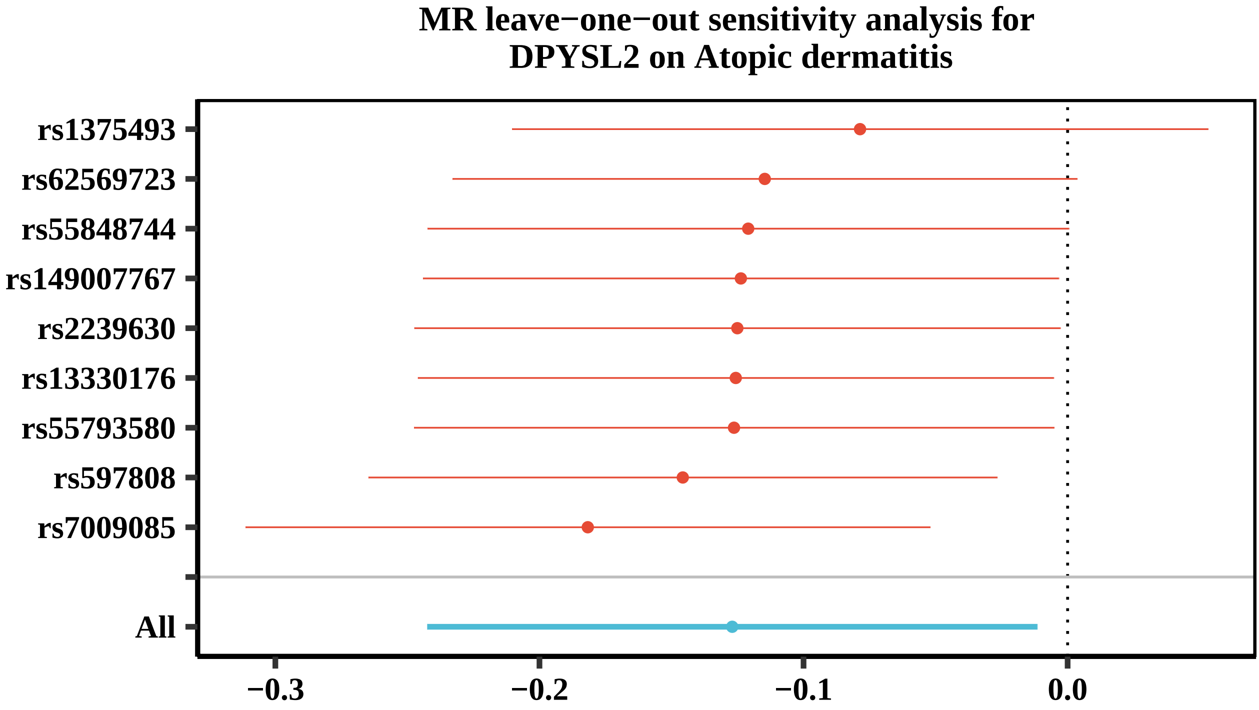
MR leave−one−out sensitivity analysis for
DPYSL2 on Atopic dermatitis
rs1375493
rs62569723
rs55848744
rs149007767
rs2239630
rs13330176
rs55793580
rs597808
rs7009085
All
−0.3
−0.2
−0.1
0.0
